# High Intensity Physical Rehabilitation Later Than 24 h Post Stroke Is Beneficial in Patients: A Pilot Randomized Controlled Trial (RCT) Study in Mild to Moderate Ischemic Stroke

**DOI:** 10.3389/fneur.2019.00113

**Published:** 2019-02-19

**Authors:** Yanna Tong, Zhe Cheng, Gary B. Rajah, Honglian Duan, Lipeng Cai, Nan Zhang, Huishan Du, Xiaokun Geng, Yuchuan Ding

**Affiliations:** ^1^China-America Institute of Neuroscience, Beijing Luhe Hospital, Affiliated to Capital Medical University, Beijing, China; ^2^Department of Neurology, Beijing Luhe Hospital, Affiliated to Capital Medical University, Beijing, China; ^3^Department of Neurosurgery, Wayne State University School of Medicine, Detroit, MI, United States

**Keywords:** acute care, ischemic stroke, early mobilization, intensity, rehabilitation

## Abstract

**Objective:** Very early mobilization was thought to contribute to beneficial outcomes in stroke-unit care, but the optimal intervention strategy including initiation time and intensity of mobilization are unclear. In this study, we sought to confirm the rehabilitative effects of different initiation times (24 vs. 48 h) with different mobilization intensities (routine or intensive) in ischemic stroke patients within three groups.

**Materials and Methods:** We conducted a randomized and controlled trial with a blinded follow-up assessment. Patients with ischemic stroke, first or recurrent, admitted to stroke unit within 24 h after stroke onset were recruited. Eligible subjects were randomly assigned (1:1:1) to 3 groups: Early Routine Mobilization in which patients received < 1.5 h/d out-of-bed mobilization within 24–48 h after stroke onset, Early Intensive Mobilization in which patients initiated ≥3 h/d mobilization at 24–48 h after the stroke onset, and Very Early Intensive Mobilization in which patients received≥3 h/d mobilization within 24 h. The modified Rankin Scale score of 0–2 was used as the primary favorable outcome.

**Results:** We analyzed 248 of the 300 patients (80 in Early Routine Mobilization, 82 in Very Early Intensive Mobilization and 86 in Early Intensive Mobilization), with 52 dropping out (20 in Early Routine Mobilization, 18 in Very Early Intensive Mobilization and 14 in Early Intensive Mobilization). Among the three groups, the Early Intensive Mobilization group had the most favorable outcomes at 3-month follow-up, followed by patients in the Early Routine Mobilization group. Patients in Very Early Intensive Mobilization received the least odds of favorable outcomes. At 3 month follow up, 53.5%, (*n* = 46) of patients with Early Intensive Mobilization showed a favorable outcome (modified Rankin Scale 0–2) (*p* = 0.041) as compared to 37.8% (*n* = 31) of patients in the Very Early Intensive Mobilization.

**Conclusions:** Post-stroke rehabilitation with high intensity physical exercise at 48 h may be beneficial. Very Early Intensive Mobilization did not lead to a favorable outcome at 3 months.

**Clinical Trial Registration:** www.chictr.org.cn, identifier ChiCTR-ICR-15005992.

## Introduction

Ischemic stroke leads to profound neurological deficits and lasting physical disability (1–4). The use of exercise-mediated adaptations to attenuate physical disability after stroke is an emerging arena in neurotherapeutics (2, 5, 6). However, fundamental questions regarding initiation time, intensity, and type of exercise, as well as other factors that affect rehabilitation remain unclear (7–9). While current guidelines recommend starting out-of-bed activity “early” during the acute phase of care, such guidelines do not specify how or if early exercise optimizes outcomes (10, 11). Many published studies have shown inconsistent results regarding the efficacy and safety of very early mobilization (VEM) after acute stroke. In a series of studies on A Very Early Rehabilitation Trail (AVERT), the authors did not recommend a certain initiation time for rehabilitation, while the study demonstrated an unfavorable outcome may be caused by a mobilization within 24 h after onset of stroke (12–15). A multicenter SEVEL (Early Sitting in Ischemic Stroke Patients) trial also did not find a significant functional improvement while initiating an early sitting protocol within 24 h after stroke onset. However, similar studies of VEM in India (16) and Japan (17), provided preliminary evidence that very early mobilization within 24 h of stroke onset was feasible, safe and cost effective The recent Cochrane systematic review of very early initiation of rehabilitation (VEI) (18) also concluded that the efficacy of VEI remains to be established. The optimal time for commencing mobilization in stroke patients remains unknown although the majority of studies address VEM. Furthermore, few studies have focused on the intensity of mobilization. The latest guidelines for management of acute ischemic stroke (10) from the American Stroke Association indicate that high-dose mobilization within 24 h of stroke onset should not be performed because it can reduce the odds of a favorable outcome at 3 months. The optimal dose of mobilization remains unknown. We surmise that an optimal rehabilitation strategy should be based on a proper combination of timing and intensity. It is highly important to understand how to rapidly and safely administer exercise after stroke. Therefore, the primary aim of our randomized controlled trial was to compare 24 h, the very early initiation time, to the 48 h, early initiation time of therapy with respect to patient outcomes. We also sought to characterize different intensities of mobilization and their relationship to functional outcomes. We sought to determine the effect of two major factors; timing and intensity, on rehabilitative outcome. Our clinical hypothesis was that intensive, early, but not too early out-of-bed activity would improve functional outcomes at 3 months. The primary outcome was to be assessed at 3 months using mRS scores.

## Materials and Methods

### Study Design and Setting

This is a single center randomized controlled trial. The study was conducted at the Stroke Unit of the Department of Neurology, Beijing Luhe Hospital, Capital Medical University, from January 1, 2015 to December 31, 2017. The institutional ethics committees approved the study. The trial was registered in the Chinese Clinical Trial Registry (ChiCTR-ICR-15005992).

### Participants

During the recruitment period, the principal investigator screened all patients admitted to stroke unit according to the following criteria.

Inclusion criteria: Patients aged 18–80 years, with a confirmed first or recurrent ischemic stroke admitted to our stroke unit within 24 h of onset, without disturbance of consciousness (score <2 for the first item of the NIHSS) and being able to react to verbal commands, were included in the study. Treatment with recombinant tissue plasminogen activator (rtPA) was allowed. Informed consent was obtained from each patient or his/her guardian before randomization.

Exclusion criteria included: (1) Premorbid disability (mRS>2); (2) Diagnosed transient ischemic attack (TIA); (3) Early acute deterioration, direct admission to the intensive care unit; (4) Any other serious medical illness or unstable coronary condition; (5) Systolic blood pressure lower than 110 mmHg or higher than 220 mmHg, oxygen saturation lower than 92% with oxygen supplementation, resting heart rate of < 40 beats per min or more than 110 beats per min, temperature < 38.5°C; (6) Treatment with thrombectomy; (7) Enrollment in another intervention trial.

The baseline characteristics of the subjects were collected at the beginning, including age, sex, stroke side, severity, and risk factors (hypertension, diabetes mellitus, ischemic heart disease, hypercholesterolemia, smoking, atrial fibrillation, previous stroke, or transient ischemic attack). Premorbid disability, admission Rankin score, rtPA treatment, daily training time and time to first mobilization after symptom onset were recorded. Physiological parameters such as temperature, heart rate, blood pressure, and saturation were also recorded twice a day as routine procedure. Neurological impairment was assessed by the 11-item National Institutes of Health Stroke Scale (NIHSS) version with a total score of 42 points (19) on admission and at discharge. The severity of the stroke was classified as mild (NIHSS score < 8), moderate (NIHSS score 8–16) or severe (NIHSS score>16) (20).

### Intervention

All patients satisfying the inclusion criteria and giving consent were randomly assigned (1:1:1) to three groups by a computer generated randomization procedure using opaque envelopes: Early Routine Mobilization Group (ERM, early but not intensive), Early Intensive Mobilization (EIM) and Very Early Intensive Mobilization (VEIM). All participants received usual standard medical care (such as anti-platelet, anti-coagulation, anti-lipidemic, anti-hypertension or anti-inflammatory injury treatment) according to their conditions.

(1) ERM Protocol- besides standard medical care, patients in this group started < 1.5 h/d (lower dose) of out-of-bed mobilization within 24–48 h after stroke onset.
(2) EIM Protocol- besides standard medical care, patients in EIM started the ≥3 h/d out-of-bed mobilization within 24–48 h after stroke onset.
(3) VEIM Protocol- besides standard medical care, patients in VEIM started the ≥3 h/d of out-of-bed mobilization within 24 h of stroke onset.

Out-of-bed mobilization included sitting, standing, and walking which were performed with or without assistance as described by the “A Very Early Rehabilitation Trial” (AVERT) Protocol (14). No special equipment was used, and mobilization included the use of standing bed and wheelchair, when necessary. All mobilization protocols were adjusted to the patients' tolerance, needs and abilities and were delivered by professional therapists or nurses. The frequency, dose, and content of mobilization varied according to physical ability and were recorded in detail by therapists or nurses. Dose monitoring was done by a specially assigned staff to ensure good compliance for this study. Physicians were asked to evaluate patients with deteriorating conditions during the exercise and to postpone mobilization when necessary. Mobilization continued for 10–14 days including the weekend.

### Outcome Assessment

The primary outcome was measured with the modified Rankin Scale (mRS) and defined as favorable mRS of 0–2 (no or minimum disability) at 3 months after stroke, while a poor outcome was defined as scores of 3–6 (moderate or severe disability, or death). Assessments during hospitalization were performed in person, or via telephone by a trained assessor at the follow-up period.

### Statistical Analysis

Sample size was estimated from our preliminary experimental results in which stroke patients were divided to two groups: very early mobilization group (within 24 h of stroke onset) and early mobilization group (24–48 h of stroke onset). Our preliminary experimental planning revealed a difference of 20% in the prevalence of patients showing a Rankin score [0–2] at 3 month after stroke onset: 35% in the very early mobilization group vs. 55% in the early mobilization group. Calculation was performed based on a type I error risk of 5% and a power of 80%, in a two-sided approach. A total of 94 patients per group was calculated as necessary to show a difference of 20% in the prevalence of patients showing a favorable outcome (Rankin score 0–2) at 3 month after stroke onset. Final planning saw the sample size adjusted to a total of 100 patients per group.

Data of all patients who completed the protocols and follow-up were analyzed and we used a Per-protocol (PP) analysis. Statistical analysis was performed using the Statistical Package for Social Science (SPSS), version 19.0 (SPSS Inc., Chicago, IL, USA). *P* < 0.05 was considered significant. Descriptive statistics were used to analyze all demographic and clinical characteristics. Continuous data was presented as mean (standard deviation) and categorical data was presented as a number and percentage. Continuous variables consistent with the normal distribution were compared by the independent samples *t*-test or analysis of variance (ANOVA), otherwise by rank sum test. Categorical variables were compared using chi-square testing.

## Results

**Figure 1:**
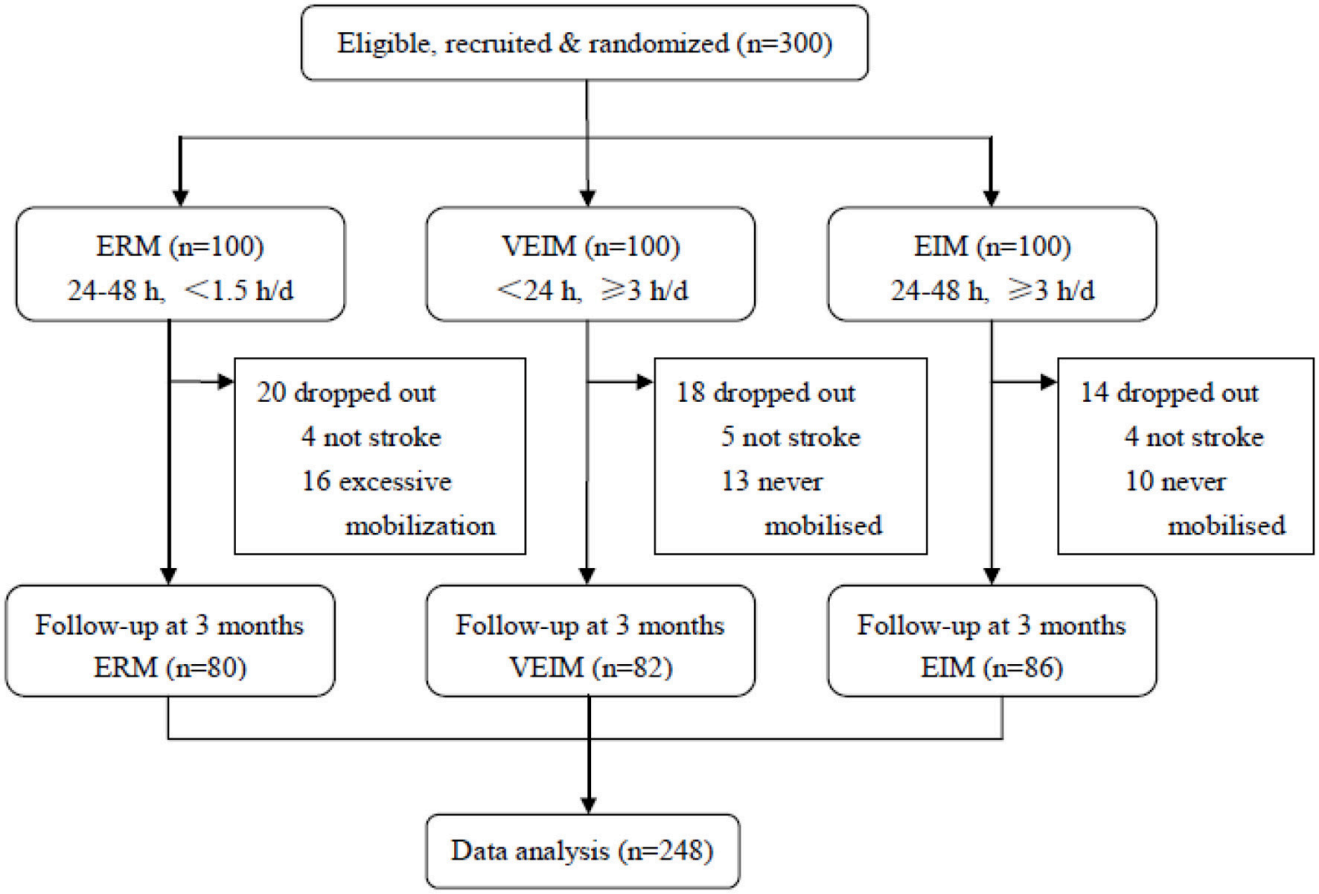
Trial profile. ERM, Early Routine Mobilization; VEIM, Very Early Intensive Mobilization; EIM, Early Intensive Mobilization. Three hundred patients were assigned randomly (1:1:1) to three groups. Two hundred and forty-eight (82.7%) patients finished the training and follow-up assessment, 80 in ERM, 82 in VEIM and 86 in EIM groups, while 52 patients (20 in ERM, 18 in VEIM, and 14 in EIM) dropped out for various reasons. In total, 80 in ERM, 86 patients in EIM and 82 patients in VEIM finished the training and the follow-up, and were thus analyzed.

From January 1, 2015 to December 31, 2017, 300 patients were assigned randomly (1:1:1) to three groups. 248 (82.7%) patients finished the training and follow-up assessment, 80 in ERM, 82 in VEIM and 86 in EIM groups, while 52 patients (20 in ERM, 18 in VEIM and 14 in EIM) dropped out for various reasons (Figure 1). Baseline characteristics including age, gender, risk factors and pre-morbid disability were similar among study groups (Table 1). The stroke severity at admission was evaluated with the NIHSS. There was no significant difference in the three groups (Table 1). Most of the patients had a first time ischemic stroke (85.0% in ERM, 79.3% in VEIM, 82.6% in EIM) and all the enrolled patients had mild or moderate strokes with NIHSS scores < 8, or between 8 and 16. Patients with NIHSS scores more than 16 were either unconscious or unable to tolerate the rehabilitation procedures. The median daily training time of patients in VEIM (184.6 min) and EIM (184.1 min) were significantly (*p* < 0.001) longer than that of patients in ERM (53.4 min), while time to first mobilization after the symptom onset was significantly (*p* < 0.001) shorter in VEIM (16.8 h) than in ERM (41.0 h) and EIM (38.0 h) (Table 2).

**Table 1:** Baseline characteristics of the patients.

	**VEIM (*n* = 82)**	**EIM (*n* = 86)**	**ERM (*n* = 80)**	***P***
Age (years)	60.2 ± 10.5 (32–80)	60.9 ± 10.7 (30–80)	62.1 ± 10.3 (39–80)	0.491
< 65	52 (63.4%)	54 (62.8%)	49 (61.3%)	0.958
65–80	30 (36.6%)	32 (37.2%)	31 (38.7%)	–
Sex (male)	67 (81.7%)	66 (76.7%)	57 (71.3%)	0.290
**RISK FACTORS**				
Hypertension	54 (65.8%)	68 (79.1%)	54 (67.5%)	0.120
Diabetes mellitus	22 (26.8%)	32 (37.2%)	32 (40.0%)	0.176
Ischemic heart disease	9 (11.0%)	13 (15.1%)	12 (15.0%)	0.679
Atrial fibrillation	5 (6.1%)	5 (5.8%)	10 (12.5%)	0.208
Hypercholesterolemia	62 (75.6%)	61 (71.9%)	49 (61.3%)	0.130
Smoking	34 (41.4%)	32 (37.2%)	27 (33.8%)	0.597
Previous stroke or TIA	17 (20.7%)	15 (17.4%)	12 (15.0%)	0.631
Pre-morbid disability				0.447
mRS 0	79 (96.3%)	82 (95.3%)	79 (98.8%)	
mRS 1	3 (3.7%)	4 (4.7%)	1 (1.2%)	
mRS 2	0	0	0	
**ADMISSION RANKIN SCORE**				
mRS 0	0	0	0	
mRS 1	12 (14.6%)	14 (16.3%)	16 (20.0%)	
mRS 2	17 (20.7%)	25 (29.1%)	16 (20.0%)	
mRS 3	21 (25.6%)	19 (22.1%)	20 (25.0%)	
mRS 4	28 (34.1%)	27 (31.4%)	26 (32.5%)	
mRS 5	4 (4.9%)	1 (1.2%)	2 (2.5%)	
mRS 6	0	0	0	
Rankin score [0–2]	29 (35.3%)	39 (45.4%)	32 (40.0%)	
**STROKE SEVERITY**				
NIHSS score	5.9 (1–16)	5.8 (1–16)	6.0 (1–16)	0.752
Mild(1–7)	58 (70.7%)	63 (73.2%)	50 (62.5%)	0.298
Moderate(8–16)	24 (29.3%)	23 (26.8%)	30 (37.5%)	
Severe(>16)	0	0		
rtPA treatment (yes)	21 (25.6%)	15 (17.4%)	20 (25%)	0.368

**Table 2:** Initiating time and intensity of mobilization.

	**VEIM (*n* = 82)**	**EIM (*n* = 86)**	**ERM (*n* = 80)**
Daily training time per person (min)	184.6 (180–220)	184.1 (180–220)	53.4 (30–90)
Time to first mobilization (h)	16.8 ± 5.2 (5–23)	38.0 ± 6.4 (25–47)	41.0 ± 4.4 (29–48)

**Figure 2:**
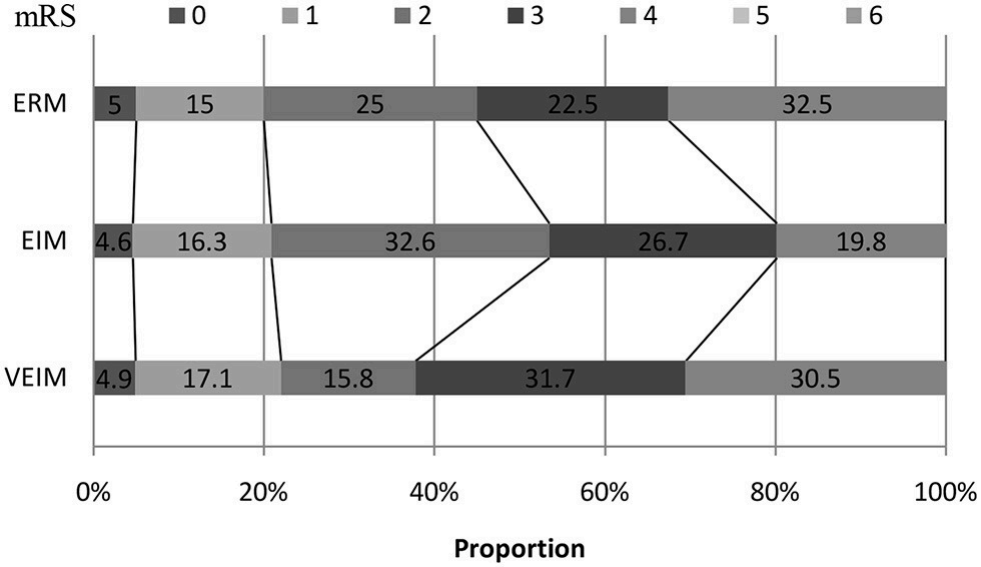
mRS shift: the percentage of patients achieving each mRS score at 3 months. ERM, Early Routine Mobilization; VEIM, Very Early Intensive Mobilization; EIM, Early Intensive Mobilization; mRS, modified Rankin Scale.

We used mRS 0–2 (minimum or no disability) for the primary favorable outcome. Although we did not see significant differences among the three groups in 3-month follow-up, the percentage of primary favorable outcomes was highest in EIM and lowest in VEIM (Figure 2 and Table 3). Furthermore, 53.5% of patients in EIM group had favorable outcomes (mRS 0–2) at 3 months, in contrast to 37.8% of the patients in VEIM, this difference was statistically significant (Table 3). In addition, more patients in EIM (53.5%) showed a favorable outcome as compared to ERM (45%), even though the difference did not reach a significant level. Taken together, EIM appeared to be the most beneficial rehabilitation program with statistically better results at 3 months, followed by ERM, while the VEIM group had the lowest positive outcomes at 3 months. mRS shift data again revealed positive functional shifts in mRS for the EIM group (Figure 2).

**Table 3:** Outcome at three months.

	**VEIM (*n* = 82)**	**EIM (*n* = 86)**	**ERM (*n* = 80)**	***p***
Favorable outcome (mRS 0–2)	31 (37.8%)	46 (53.5%)	36 (45%)	0.041 (VEIM vs. EIM)
				0.353 (VEIM vs. ERM)
				0.274 (EIM vs. ERM)
mRS Category 0	4 (4.9%)	4 (4.6%)	4 (5%)	
1	14 (17.1%)	14 (16.3%)	12 (15%)	
2	13 (15.8%)	28 (32.6%)	20 (25%)	
3	26 (31.7%)	23 (26.7%)	18 (22.5%)	
4	25 (30.5%)	17 (19.8%)	26 (32.5%)	
5	0	0	0	
6	0	0	0	

## Discussion

In the present study, we identified that more patients with utilization of the early (24–48 h) intensive mobilization (EIM) program received a favorable functional outcome, as compared to early (24–48 h) but not intensive (routine) mobilization (ERM), although the difference was not statistically significant. We then confirmed that EIM was better able to improve functional outcomes than VEIM at 3 months. In contrast to EIM, VEIM showed a poorer outcome overall at 3 months. A higher intensity but not too early mobilization appeared most beneficial in our study for rehabilitation after acute stroke.

As compared to AVERT (12–15), the pioneering studies in the realm of very early mobilization, the present study shared several similarities, although our study was a single center study and had a relatively small sample size. Our study was randomized and controlled, and the study duration was up to 14 days. We used same definition of “very early mobilization” for out-of-bed interventions commenced within 24 h after stroke, the same interventions for out-of-bed mobilization, and the same outcome measure (mRS scores). Very comparable results were observed in the higher dose (≥3 h/d out-of-bed mobilization), very early mobilization protocol (initiating within 24 h post stroke) in both studies. This mobilization protocol was associated with a reduction in the odds of a favorable outcome at 3-months follow-up, despite the 2011 AVERT follow-up suggesting VEIM may fast track ambulation recovery (21). It was not until the final 2015 AVERT results published in Lancet did the VEIM group results change suggesting worse outcomes at 3 months for this group (15). Thus, the question of the therapeutic efficacy of VEIM was left unsettled. Our results seem to confirm this therapy is not useful.

Importantly, as compared to AVERT, the present study was unique as follows: (1) we directly compared very early (within 24 h) and early (24–48 h) mobilization with the same intensive mobilization (≥3 h/d); (2) we directly compared routine (< 1.5 h/d) and intensive (≥3 h/d) mobilization with same early initiation (24–48 h). In addition, the protocol of intensity in our trial was described in more details. In AVERT, the concept of intensity mobilization in intervention group (VEM) was blurred as it was just double the control group dose, without a specific amount of daily training time duration. A retrospective cohort study (22) consisting of 360 patients demonstrated that subjects who received >3.0 h of therapy daily made significantly more functional gains than those receiving < 3.0 h daily. Therefore, we used this duration for intensive mobilization and 1.5 h per day for routine mobilization.

AVERT found the higher dose, very early mobilization protocol was associated with a reduction in the odds of a favorable outcome (modified Rankin Scale [mRS] 0–2) at 3 months (15). One notable limitation of this trial is that most patients (roughly 60%) in usual care group started out-of-bed therapy within 24 h of stroke onset, rather than more than 24 h as it was designed (15). As a result, the difference between the intervention and control groups regarding initiation time for mobilization, though statistically significant, was small—mean 18.5 vs. 22.4 h. However, the difference in intensity between the two groups was significant, with the intervention group spending almost three times longer out of bed than controls (201.5 vs. 70 min). Given this, the difference in intensity probably played a greater role on outcomes than the difference in initiation time in AVERT. Our study sought to rectify this conundrum and determined the factor of initiation time with the same intensity and the factor of intensity with the same initiation time on outcome at 3 months after stroke. With significantly different initiation times between the two groups (EIM 38.0 h vs. VEIM 16.8 h) and almost the same intensity (EIM 184.1 min vs. VEIM 184.6 min), we found patients in EIM had significantly greater odds of favorable outcomes than patients in VEIM. Obviously, the difference in initiation time played a unique role in the outcomes of our study.

In order to better understand whether very early rehabilitation is beneficial or harmful, it is important to assess physiologic and animal studies. Given the labile blood pressure in the peri stroke period (23), very early mobilization could reduce cerebral blood flow and harm the ischemic penumbra (24). This maybe related to head position and redistribution of blood to other organs, especially standing musculature. Furthermore, the poor outcome caused by very early and intensive mobilization may be related to a disturbed auto regulatory regional cerebral blood flow (rCBF). Under physiologic conditions, the cerebral auto-regulation mechanisms keep the cerebral blood flow (CBF) relatively stable. During acute stroke, the cerebral auto-regulation mechanisms are impaired and any fluctuation in blood pressure can affect the CBF directly (25). Moreover, recent research indicates that moderate exercise is associated with an increase in cerebral blood flow (CBF). Increases in exercise intensity up to 60% of maximal oxygen uptake elevated CBF (26–28). If more than that level, CBF was decreased despite the increased cerebral metabolic demand during early and intensive exercise in VEIM, possibly acting as an independent harmful influence on cerebral function (27, 28). Krakauer and colleagues (29) considered that too early mobilization of the affected limbs after brain injury may hamper brain plasticity as it may weaken GABA-mediated tonic inhibition. Reducing GABA-mediated inhibition in the first few days after stroke onset may enlarge the infarct size (29).

Several animal experiments support the notion that very early rehabilitation is not beneficial (30–32). Exercise training in rats beginning at 24 h post-stroke was associated with enlargement of ischemic lesions compared with animals who began training at 7 days (30). Shen et al. (31) found that hyperglycolysis and activation of nicotinamide adenine dinucleotide phosphate oxidase (NOX) was associated with an elevation in apoptotic cell death. This was increased in rats after very early exercise (6 h-24 h), but not after late exercise (3 days). Li et al. (32) found that inflammatory cytokines were increased at 6 h but not at 24 h or 3 days with exercise in rats, and apoptotic cell death was enhanced by very early exercise in association with increased expression of pro-apoptotic proteins. Although correlative age data between rats and humans may be imperfect, a study has suggested that 24 h for an adult rat corresponds to 30 days for an adult human (33). It raises possibility that a 24 h exercise implementation in rats would simulate human conditions at a latter time point in rehabilitation. Furthermore not all rat studies have shown exercise therapy at 24 h to be harmful, Zhang et al. (34) reported smaller tissue infarct sizes and improved outcomes.

In the present study, the patients recruited were not representative of the whole stroke population since patients with severe aphasia, disturbance of consciousness or thrombectomy were excluded. As our study was conducted at one center, we did not recruit enough patients (100 in the 3 groups) to fulfill our initial Power Assessment. However, the relatively small sample size and single-center nature of the study have nevertheless suggested a meaningful conclusion that high intensity physical rehabilitation later than 24 h post stroke is beneficial in patients. The beneficial effects of early but not too early with intensive mobilization protocol warrant a future randomized and controlled multicenter trial study with a larger sample size. We also confirmed the previous findings of the AVERT study that VEIM therapy should not be used outside of a randomized trial.

## Author Contributions

YT, GR, LC, and NZ performed the study, analyzed data, and prepared the manuscript. YD, HoD, XG, and GR designed the study and revised the manuscript. YT, ZC, and HuD evaluated the subjects.

### Conflict of Interest Statement

The authors declare that the research was conducted in the absence of any commercial or financial relationships that could be construed as a potential conflict of interest.
